# Mapping knowledge structure and research trends of knee osteoarthritis with meniscus in two decades: A bibliometric analysis

**DOI:** 10.3389/fsurg.2022.939003

**Published:** 2022-10-28

**Authors:** Weijian Chen, Yaqin Yang, Gangjian Tang

**Affiliations:** ^1^ Graduate College, Guangxi University of Chinese Medicine, Nanning, China; ^2^ Department of Orthopedics, Guilin Hospital of Traditional Chinese Medicine, Guilin, China; ^3^ The Second Clinical Medical College, Guangzhou University of Chinese Medicine, Guangzhou, China; ^4^ Department of Nephrology, Guangdong Provincial Hospital of Chinese Medicine, Guangzhou, China

**Keywords:** knee osteoarthritis (KOA), meniscus, bibliometrics [MeSH], visualized study, research trends

## Abstract

**Background:**

Knee osteoarthritis (KOA) is a chronic degenerative disease that is closely related to the meniscus. Currently, no bibliometric studies have jointly analyzed KOA and the meniscus. This study aimed to provide a comprehensive analysis of the knowledge structure of KOA and the meniscus across two decades and to identify the emerging research trends from a bibliometric perspective.

**Methods:**

All articles reporting KOA and the meniscus from 2001 to 2021 were obtained from the Web of Science Core Collection. R software, CiteSpace, VOS Viewer, and Microsoft Excel were used to analyze the publications including the authors, cited authors, journals, cited journals, country of research, institutions, and research focus. These data were used to generate visual knowledge maps of the outputs.

**Results:**

A total of 3,218 articles were retrieved. Guermazi was identified as the author who had contributed the most to the field and *Osteoarthritis and Cartilage* was identified as the most productive research journal. The United States is the global leader in the field and the center for international cooperation with less international collaboration occurring in Eastern Asia. Boston University was the most prolific institution. According to the data, “articular-cartilage,” “meniscectomy,” “follow-up,” “anterior cruciate ligament,” and “cartilage” were identified as research hotspots in the field. “Consequences,” “prognostic-factors,” and “receptor” were predicted as future hot topics of research.

**Conclusions:**

This study is the first comprehensive bibliometric study to jointly analyze KOA and the meniscus. Our data enable a better understanding of research trends and identify research hotspots and gaps in knowledge across the field. Our findings provide practical information for researchers to better understand the key research areas and identify the research frontiers and future hot topics.

## Introduction

Knee osteoarthritis (KOA) is a common chronic degenerative disease that is often associated with synovitis and progressive cartilage injury that is characterized by joint pain, stiffness, and dysfunction. Approximately 22% of the general population suffer from KOA-associated pain and the condition is more common in the elderly (1). The meniscus is a critical part of the knee joint that is attached by ligaments to provide joint congruity. The meniscus functions to distribute mechanical loads on the articular cartilage and lubricates the joint with a fluid film and proteoglycans (2). KOA and the meniscus are areas of intense research interest with several thousands of research articles published in this area every year.

The relationship between KOA and the meniscus is complex as meniscus injury can be both a cause and consequence of KOA. Medial meniscus root tear is increasingly considered a common cause of joint-line pain and early onset of KOA (3). A cohort study of young adults found that meniscus tears greatly increase the risk of KOA (4). Also, a previous study showed that many patients with meniscus injuries develop radiologic signs of osteoarthritis (OA) at a young age (5).

Bibliometric analysis is a literature-based analysis framework that is used to identify overlooked connections between disparate papers (6). Over the past two decades, several KOA-related or meniscal bibliometric papers have been published that include acupuncture (7) and surgical treatments (8–13). However, to the best of our knowledge, no study has jointly analyzed the fields of KOA and meniscus together. This study aimed to identify the research trends and hot topics in KOA and meniscus research by analyzing published papers internationally in the past 20 years. We also briefly discussed previous research including the high-yielding authors and their research teams to provide a reference for further learning in the field.

## Materials and methods

### Data acquisition and retrieval strategies

This bibliometric study was based on the Clarivate Analytics’ Web of Science, which is one of the most optimal and comprehensive database to retrieve bibliometric indicators and academic information. In this study, all the documents regarding the KOA and meniscus were retrieved and downloaded from the Web of Science Core Collection (WoSCC) database with the following search strategy: (TS = (knee osteoarthritis) OR TS = (knees osteoarthritis) OR TS = (osteoarthritis of knee) OR TS = (osteoarthritis of knees) OR TS = (Osteoarthritis, Knee) OR TS = (Osteoarthritis, Knees)) AND TS = (meniscus OR meniscal OR menisci). Since the articles published in 2022 are too few to show the trend, the time frame of data was set from 2001 to 2021. The publication's language was not limited. In addition, the article types including editorials, case reports or case series, reviews, meta-analyses, conference abstracts, and news reports were excluded. As a result, a total of 3,218 articles were found and sent to CiteSpace V and R software.

### Data analysis

Statistical analysis was performed using R software v3.6.3 and Microsoft Excel 2019.

Visualization analyses were conducted using R software v3.6.3, CiteSpace v5.8R3, and VOS viewer. CiteSpace is an optimal visual analysis software based on Java and developed by Synnestvedt et al. (14). In this study, CiteSpace was used to identify the cited journals and cited authors. The map created in CiteSpace is composed of data rings and lines. The data rings reflect the corresponding occurrence or citation frequencies of the analyzed papers. The color of the rings represents the citation time, and the width of the ring is proportional to the corresponding period citation number. The line between the rings represents associations such as the cited relationship and the width of the line conforms to the intensity of cited.

VOS viewer is a free software that can be used to analyze the relationships between co-authors, highly cited references, and for co-citation network analysis (15). VOS viewer has text mining capabilities that were used in this study to analyze co-authorship. An online bibliometric platform (https://bibliometric.com/) was used to perform collaboration analysis across different countries.

## Results

### Analysis of annual publications and publication trends

**Figure 1:**
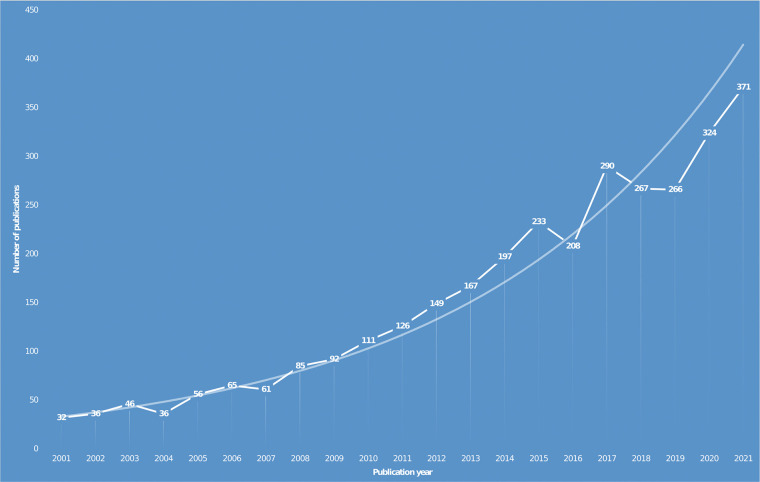
Annual research publications involving KOA and the meniscus from 2001 to 2021. KOA, knee osteoarthritis.

Keyword search in WoSCC identified a total of 3,218 publications that were included in this study. The specific number of articles published each year is shown as a line chart with a trend line in Figure 1. The number of articles on KOA and the meniscus in the past two decades showed a general increasing trend with slight fluctuations. At the beginning of the 21st century, there were few studies on KOA and the meniscus indicating a low level of interest in this research area. After this period, the number of papers grew steadily until 2015. In 2016, the number of papers decreased from 233 to 208 in 2016 but then increased rapidly to 290 in 2017. From 2018 to 2021, the number of papers was lower than the general trend line. However, the growth rate followed the forecast trend line indicating that the research studies on KOA and the meniscus were increasingly more common.

### Analysis of authors and cited authors

**Figure 2:**
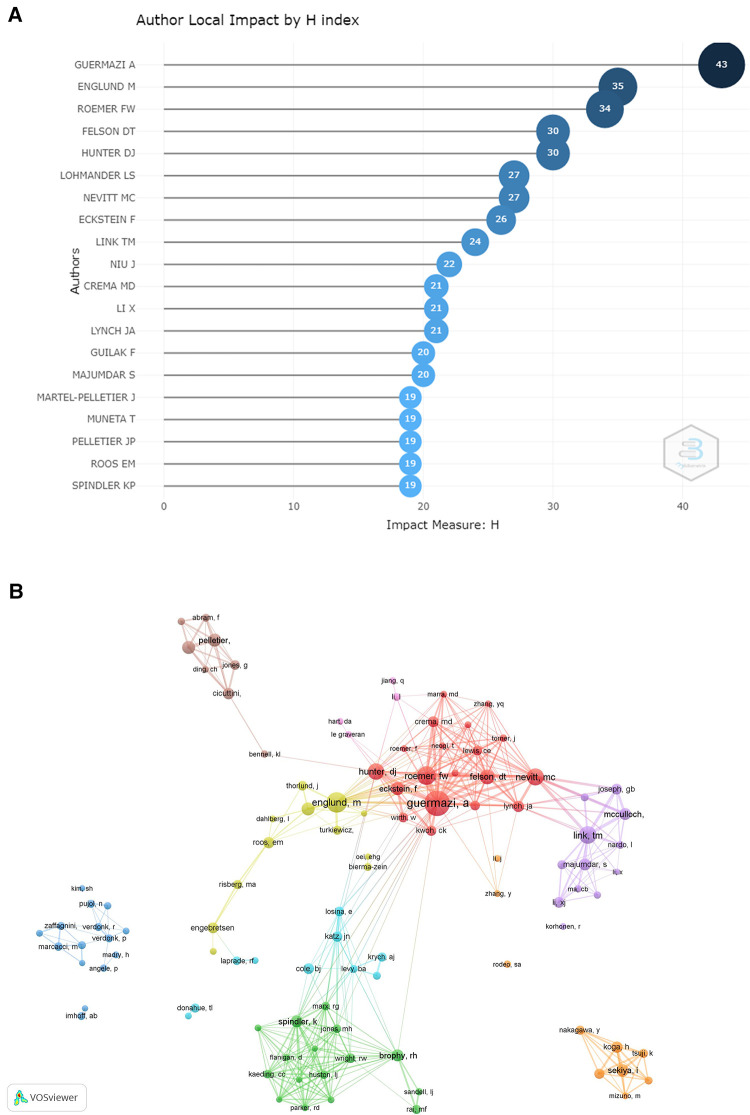
(**A**) The authors with the highest H-index. (**B**) The authors’ co-authorship visualization.

The number of scientific articles written by one scholar can indicate the extent of research activity and contribution in the field. The top 20 authors of KOA with meniscus are shown in Table 1. From the perspective of articles counts, Guermazi (126) was the most contributive author followed by Englund (101) and Roemer (83). The authors with the highest H-index are shown in Figure 2A. It was found that the top three with the highest number of publications also occupied the top three positions for the highest H-index.

**Table 1:** The top 20 authors with the most papers and citations.

Authors	Articles	Author	Citations
Guermazi A	126	Englund M	763
Englund M	101	Hunter DJ	520
Roemer FW	83	Felson DT	511
Hunter DJ	71	Kellgren JH	472
Link TM	64	Lohmander LS	412
Eckstein F	52	Roos EM	359
Nevitt MC	52	Peterfy CG	320
Felson DT	50	Roos H	310
Li X	49	Noyes FR	304
Pelletier JP	48	Glasson SS	267
Lohmander LS	43	Roemer FW	246
Martel-Pelletier J	41	Eckstein F	233
Brophy RH	40	Shelbourne KD	215
Majumdar S	36	Sharma L	210
Roos EM	36	Berthiaume MJ	192
Crema MD	35	Guermazi A	190
Katz JN	35	Loeser RF	186
Spindler KP	35	Oiestad BE	179
Wang Y	35	Fairbank TJ	173
Koga H	34	Sihvonen R	154

Every researcher is an expert in different research priorities, having unique professional knowledge. Cross-cooperation can generate creative ideas and promote research subject productivity. Moreover, analyzing the co-authorship of authors is advantageous for researchers to understand existing partnerships and develop potential cooperative research. In Figure 2B, an overlay visualization map, generated by VOS viewer, revealed the author's co-authorship analysis. It can be found that several research clusters were generated and every cluster had a strong link with one or two core researchers such as Guermazi, Englund, Link, and Sekiya. Roemer was a member of Guermazi's team. Englund works closely with the team. In addition, the researchers in Japan collaborated closely, but not much with researchers from other countries.

**Figure 3:**
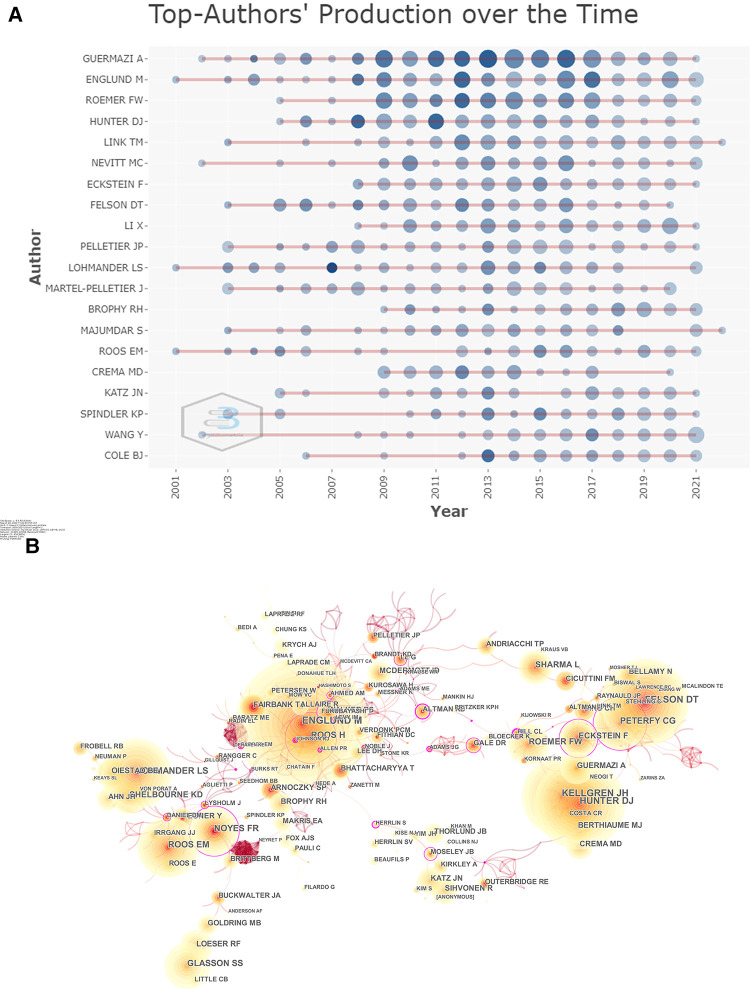
(**A**) The information of high-yielding authors’ production over the time. (**B**) Map of cited authors’ analysis related to KOA with meniscus.

The achievement paths of high-yielding authors in different periods are generated in Figure 3A; it can be concluded that the majority of authors began to express interest in the field around 2003, and Guermazi has plowed deep into this field for many years since 2002, whose output is concentrated in 2009 to 2017. The research results from Malyszko, Wolf, and Li had been updated until recently. Figure 3B shows the authors map by CiteSpace. The top five authors with the most citations were Englund (763), Hunter (520), Felson (511), Kellgren (472), and Lohmander (412), and the list is in Table 1.

### Analysis of the journals and cited journals

For decades, scientific articles have always been essential carriers for scientific exchanges of scientists and researchers in every field. The publication of research data and results in a peer-reviewed journal is an integral component of establishing continuous scientific communication. The top 20 journals are listed in Table 2. Of these journals, *Osteoarthritis and Cartilage* published the most articles (305), followed by *Knee Surgery Sports Traumatology Arthroscopy* (240) and *American Journal of Sports Medicine* (225). The 2020 Impact Factor (IF) ranged from 19.103 (*Annals of the Rheumatic Diseases*) to 2.119 (*Skeletal Radiology*).

**Table 2:** Top 20 journals with most articles in KOA with meniscus research.

Sources title	Articles	Percent	IF 2020	Category Quartile 2020
*Osteoarthritis and Cartilage*	305	9.5%	6.576	Q1
*Knee Surgery Sports Traumatology Arthroscopy*	240	7.5%	4.342	Q1
*American Journal of Sports Medicine*	225	7.0%	6.203	Q1
*Journal of Orthopaedic Research*	109	3.4%	3.494	Q1
*Arthroscopy—The Journal of Arthroscopic And Related Surgery*	103	3.2%	4.772	Q1
*Knee*	86	2.7%	2.199	Q3
*Orthopaedic Journal of Sports Medicine*	68	2.1%	2.727	Q2
*BMC Musculoskeletal Disorders*	63	2.0%	2.355	Q3
*Arthritis and Rheumatism*	53	1.6%	—	—
*Journal of Knee Surgery*	48	1.5%	2.757	Q2
*Arthritis Research & Therapy*	47	1.5%	5.156	Q2
*Journal of Biomechanics*	45	1.4%	2.712	Q3
*Annals of the Rheumatic Diseases*	44	1.4%	19.103	Q1
*Cartilage*	40	1.2%	4.634	Q1
*Scientific Reports*	40	1.2%	4.38	Q1
*Skeletal Radiology*	40	1.2%	2.199	Q3
*Arthritis & Rheumatology*	36	1.1%	10.995	Q1
*Journal of Bone and Joint Surgery—American Volume*	36	1.1%	5.284	Q1
*Archives of Orthopaedic and Trauma Surgery*	34	1.1%	3.067	Q2
*PLoS One*	33	1.0%	3.24	Q2

KOA, knee osteoarthritis; IF, Impact Factor.

Journal citation analyzes the journals in which the references are in. It also can be used to discover hot journals, the latest literature result tracking, and manuscript publishing. Table 3, generated by CiteSpace, reveals the citations of journals in the field of meniscus studies on KOA. We found that *Osteoarthritis and Cartilage* had the highest citation frequency (2,228), followed by *American Journal of Sports Medicine* (1,949) and *Journal of Bone And Joint Surgery-American Volume* (1,820).

**Table 3:** The most citations of journals in meniscus with KOA.

Source	Cited frequency
*Osteoarthritis and Cartilage*	2,228
*American Journal of Sports Medicine*	1,949
*Journal of Bone and Joint Surgery—American Volume*	1,820
*Arthritis and Rheumatism*	1,637
*Clinical Orthopaedics and Related Research*	1,586

### Analysis of most productive countries and institutions

**Figure 4:**
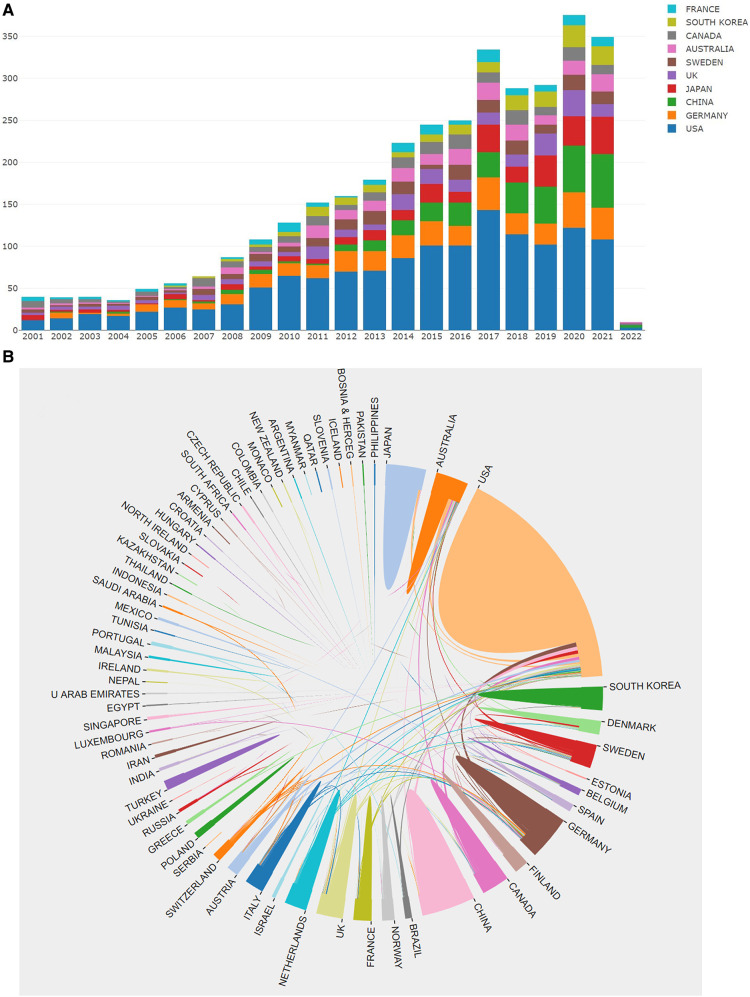
(**A**) The annual number of publications by countries. (**B**) International collaboration analysis among different countries.

**Figure 5:**
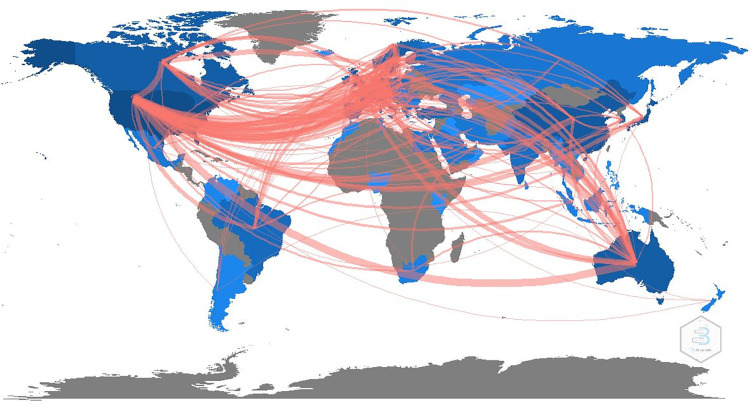
The world map shows the contribution of every country based on paper counts.

Publication analyses were based on countries and institutions in this section. In specific, as displayed in Table 4 and Figures 4, 5, the United States ranked first with 5,119 times, followed by China (1,352), Germany (1,011), Japan (924), and Australia (647). The top five most cited countries include the United States (46,157), Sweden (9,204), Germany (5,649), the United Kingdom (5,392), and Australia (5,034).

**Table 4:** Top 20 countries with the highest frequency in all authors and citations.

Country	Frequency	Country	Citations
United States	5,119	United States	46,157
China	1,352	Sweden	9,204
Germany	1,011	Germany	5,649
Japan	924	UK	5,392
Australia	647	Australia	5,034
UK	636	Canada	4,352
Canada	610	Japan	4,153
South Korea	579	Netherlands	3,289
Sweden	496	China	3,277
Netherlands	414	Korea	3,107
Italy	410	France	2,586
France	409	Norway	2,574
Finland	268	Italy	2,514
Denmark	258	Belgium	2,043
Norway	224	Finland	1,499
Turkey	212	Austria	1,386
Austria	205	Denmark	1,349
Switzerland	179	Spain	985
Spain	149	Turkey	897
Belgium	138	Switzerland	825

As can be seen from Figures 4, 5, this study used R software to create the country collaboration network map, which indicated the international collaboration among countries. The line thickness among countries reveals the intensity of cooperation. It was found that the United States had the most wide-ranging collaboration and a great number of cooperating countries, followed by Australia, Denmark, Sweden, and Germany. In contrast, China, South Korea, and Japan were less cooperative. Overall, most of the partnerships were mainly confined to Europe, America, and Australia. Cooperation in East Asia and other countries had the potential to be further enhanced.

Prolific institutions in publishing papers on the field of KOA with meniscus are presented in Table 5. Among the top 20 most active institutions, 12 were in the United States, 2 were in Australia, 2 were in Japan, and the remaining 4 were, respectively, from Sweden, Canada, Denmark, and Germany. To be specific, Boston University in America was the most active institution with 374 articles. University of California, San Francisco, in the United States was in the second place with 329 publications, while Lund University in Norway acquired third place with 234 publications. Among these 20 institutions, with the exception of Hospital for Special Surgery, the only medical institution, the other 19 are universities.

**Table 5:** The article number of the top 20 most high-yielding institutions.

Affiliations	Articles
Boston University	374
University of California, San Francisco	329
Lund University	234
Duke University	192
Washington University	173
University of Sydney	166
Rush University	161
Hospital for Special Surgery	158
Stanford University	152
University of Calgary	142
Tokyo Medical and Dental University	141
The Ohio State University	120
University of Pittsburgh	111
University of Southern Denmark	108
Harvard University	99
The University of North Carolina System	96
Monash University	85
University of Missouri	84
Juntendo University	82
Technical University of Munich	82

### Analysis of keywords and trend topics

**Figure 6:**
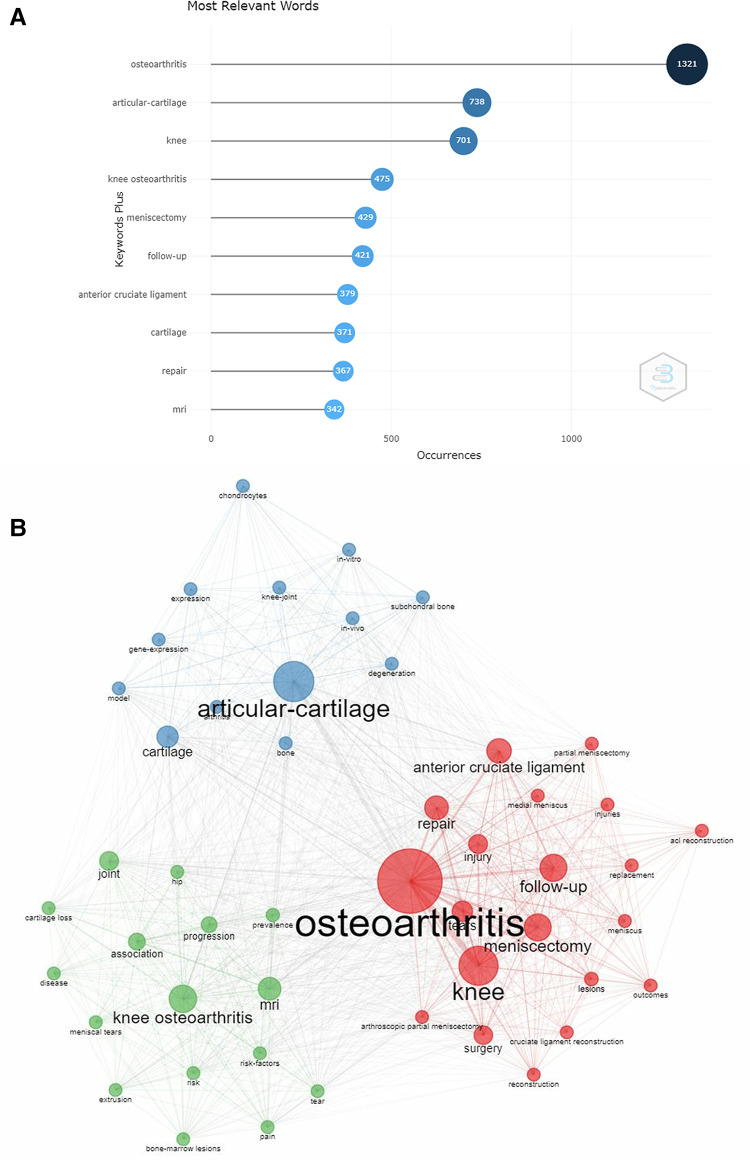
(**A**) Keywords based on 20-year publications. (**B**) The visualization map of popular keywords.

**Figure 7:**
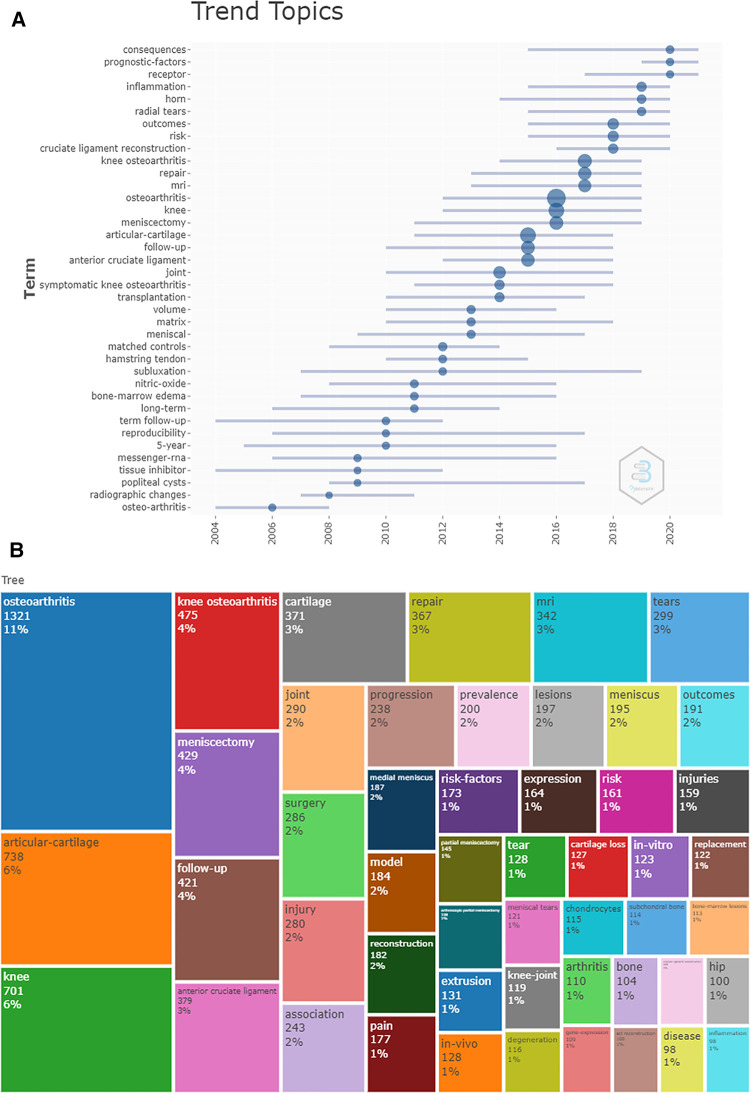
(**A**) The map of trend topics over the time. (**B**) Visualization of the proportion of keywords.

It was believed that the increased number of keyword burst and frequency within a certain period are the indicators for evaluating the most cutting-edge themes and revealing the emerging trends. According to Figure 6A, removing “osteoarthritis,” “knee osteoarthritis,” and “knee,” we found that the popular keywords are “articular-cartilage,” “meniscectomy,” “follow-up,” “anterior cruciate ligament,” “cartilage,” “repair,” and “MRI” (magnetic resonance imaging). Based on our clinical experience, the keyword “repair” means “meniscal repair.” Observing the relationship between keywords, we can further understand the research direction of segmentation. As shown in Figure 6B, popular keywords can be summarized into the three research hot spots. Displayed in red, injury, surgery, meniscus, and repair were closely related to the theme word “osteoarthritis.” The theme “articular-cartilage” in blue, involving the field of gene expression, chondrocytes, and model, was mainly focusing on the tissue, cell, and gene-level study. The keywords in green are clinically relevant, including risk factors, prevalence, MRI, tear, and so on. A topics trend is provided in Figure 7A. The trend words “consequences,” “prognostic-factors,” and “receptor” were the latest research focus; the trend topics of every period can also be found. Figure 7B intuitively displays the proportion of each keyword through the area size.

## Discussion

In this review, a total of 3,218 studies were used to identify the research trends in KOA and the meniscus. In the past two decades, the annual number of publications has significantly increased indicating the rapid progression of research involving KOA and the meniscus. This trend may be due to the progress in surgical techniques and clinical outcomes that have led to the exploration of interactions between structures in the knee joint.

From the perspective of research and citation counts, Guermazi, Englund, and Roemer were the top three authors in the field that had the most papers and the highest H-indices. Guermazi and Roemer were in the same research team. Guermazi has published many studies using MRI, plain radiography, and scoring systems. His colleagues applied 3D-MRI technology to measure longitudinal changes in the meniscus in KOA. The 2-year follow-up exam showed that the tibial plateau had significantly decreased coverage by the medial and lateral meniscus (16). In the latest article up to 2021, a new OA severity score was proposed based on MRI (17). This score was based on the whole-joint and included meniscus scoring. A study from Roemer proposed that 4 mm or more is the optimum cut-off for meniscal body extrusion on knee MRI relative to the widely acknowledged standard (18). The use of MRI in the Osteoarthritis Initiative led Englund to conclude that meniscal extrusion was associated with knee pain in possible early-stage KOA development (19). Since the top three authors also had the highest h-indices, when scholars want to search for valuable articles, their articles may warrant prior consideration. Also, since the vast majority of their articles were published in 2009–2017, the progress in the field may be reported more recently by other productive authors.

Analysis of the distribution of journal sources allows scholars to quickly select the most appropriate journals for their articles. According to Journal Citation Reports (JCR), around 70% of the journals were classified as orthopedics or sports science, or surgery. Around 25% of journals were listed as rheumatology. *Osteoarthritis and Cartilage* was the first in the list that ranked second in orthopedics and sixth in the rheumatology category of JCR. In addition, 50% of the journals belonged to JCR Q1. Except for the number of papers, the cited frequency also represents the influence of the journal in the field. Our data indicated that numerous high-profile studies were published in these journals.

The most productive and most cited countries were very similar. China and Japan were in the top five most productive countries representing the high level of research activity in Eastern Asia. However, the top five most cited countries were almost all from Europe, Australia, and the United States. The subtle difference reflects that the research from these countries received a higher level of recognition. Cooperation among different units was found to be conducive to multidisciplinary integration and the United States had the highest level and range of cooperation with much lower levels of collaboration in Asian countries. The strengthening of cross-country cooperation may be a way for Asian countries to enhance the quality and citation frequency of their research articles.

Prolific institutions that contribute a considerable amount of output are found in more productive countries. For example, three-fifths of the institutions in Table 4 are located in the United States. Some countries that have high productivity, such as China, lack productive institutions. This may reflect the current situation of scattered research.

Knee joint structures appeared frequently in the keywords and trending topics, indicating a close relationship between various knee injuries. Meniscectomy is one of the most common operations for the treatment of meniscus injury (20). Although it is considered the treatment for irreparable and symptomatic meniscus tears, meniscectomy lacks obvious clinical benefits (21). A meta-analysis in 2018 found that total meniscectomy was associated with higher rates of osteoarthritis, total knee replacement, and higher costs compared to meniscus repair and nonoperative treatments for medial meniscus root tears (3). Recently, arthroscopic partial meniscectomy (APM) has been established in the clinic; however, its use remains controversial. APM can effectively reduce pain levels and improve knee function (22). Also, compared to other treatments, APM is expensive and can accelerate cartilage degeneration to increase the risk of KOA (23).

Meniscus saving has become a prolific research theme and the field has shifted toward meniscal suture repair. Meniscus repair delays the degeneration of the joint and KOA by reducing mechanical changes and has similar short-term outcomes to meniscectomy with a better long-term prognosis (24). Increasing evidence has led to changes in opinion such as the broad understanding that white–white tears are usually dislodged, whereas red–white and red–red tears are often repaired (25). In 2019, Cinque et al. reported that white–white repairs could also have higher functional scores than preoperative ones (26). In addition, complications associated with different repair techniques are being discussed and are leading to improvements in the latest field. A study showed that internal meniscal repair had a lower rate of nerve injury compared to the inside-out approach (27). Further analysis showed that the inside-out approach was better as it had a lower risk of tear propagation and cartilage injury (28). A systematic review found that 64.7% of articles tested medical devices and three surgical advances, especially, cross-suture, rebar, and transtibial tunnels were detailed (25). Finally, biologics may become established as a future strategy for meniscus repair yet it has not been fully explored in the clinic.

The anterior cruciate ligament (ACL) functions to maintain the stability of the knee joint by controlling the normal static and dynamic loads on the knee. The treatment of meniscus and its relevance have always been a focus of research. A consensus statement stated that the treatment decisions for ACL should consider other supporting structures including the meniscus and cartilage (29). Biomechanical evaluations showed no significant differences in knee kinematics between ACL reconstruction (ACLR) with concomitant medial meniscal repair and an intact knee (30). Conversely, a 2-year follow-up reported that isolated ACLR had similar outcomes compared to the group with meniscus repair and was even better than meniscectomy (31). Also, an interesting study with an 18-year follow-up found that acute ACLR within 6 months significantly reduces the rate of secondary meniscal tears rate compared to delayed ACLR and nonoperative management (32). In conclusion, ACL treatment strategies have been developed but require further clinical evaluation.

Articular cartilage degeneration caused by cartilage and meniscus injury is a direct cause of KOA. Meniscus and ligament tears contribute to the development of KOA as the average joint loading is changed. Also, KOA and meniscus injury often appear simultaneously (33). Farina et al. indicated that preoperative mechanical knee symptoms that are presumed to relate to specific meniscal pathology, in fact, are strongly associated with cartilage damage (34). Lysosomes are known to impair autophagy in KOA cartilage (35). Joseph et al. demonstrated positive associations between serum biomarkers including sMMP3 and sCOMP, and cartilage T2 in MRI. This study demonstrated the associations between these biomarkers and cartilage extracellular matrix (36). In cartilage repair therapy, tissue engineering strategies are research hot spots. Osteochondral autograft transfer offers improved load-bearing ability, and a variety of scaffold and scaffold-free methods have been used to advance engineering techniques to provide long-term solutions (33).

In the early years, MRI results, meniscal damage, displacement, joint injury, and risk factors were the most common studies areas of KOA with the meniscus (37, 38). Englund and Lohmander evaluated patients who had undergone previous meniscal resection and found that degenerative meniscus tears and lateral meniscectomy were frequently associated with radiographic KOA (39). Until recently, a large number of articles have been published based on age, sex, risk factors, meniscus extrusion, and KOA prediction.

## Conclusion

This study comprehensively analyzed the studies on KOA and meniscus published between 2001 and 2021 utilizing R software, VOS Viewer, CiteSpace, and Microsoft Excel. The relative research interest and number of publications were globally increasing per year. Guermazi, Englund, and Roemer are the top authors with the most articles and highest H-index. *Osteoarthritis and Cartilage* is the journal that owns the most article number and cited frequency. The United States and Boston University are, respectively, the most productive country and institution. Research topics mainly involved articular cartilage, meniscectomy, follow-up, anterior cruciate ligament, repair, and MRI. Consequently, prognostic factors and receptor were potential frontiers for research.

In summary, this study was the first to review meniscus and KOA by bibliometric analysis in the past two decades, which provides a reference for future studies.

## Limitations

The study had several limitations. First, this paper only focused on the WoSCC database, which may not be comprehensive enough. This study chose only one database for two reasons. On the one hand, WoSCC was the most commonly used database in bibliometric analysis. On the other hand, software like CiteSpace cannot analyze the content from multiple databases. Second, discrepancy between our analysis results and real-world conditions may exist. For instance, the influence of recently published high-quality publications may not be emphasized because of their low current citation numbers.

## Data Availability

The original contributions presented in the study are included in the article/Supplementary Material, further inquiries can be directed to the corresponding author.
